# Illness perception, diabetes knowledge and self-care practices among type-2 diabetes patients: a cross-sectional study

**DOI:** 10.1186/s13104-017-2707-5

**Published:** 2017-08-10

**Authors:** Nuworza Kugbey, Kwaku Oppong Asante, Korkor Adulai

**Affiliations:** 1 Department of Family and Community Health, School of Public Health, University of Health and Allied Sciences, Hohoe, Volta Region Ghana; 2 Department of Psychology, School of Social Sciences, University of Ghana, Legon, Accra, Ghana; 3 Discipline of Psychology, School of Applied Human Sciences, University of KwaZulu-Natal, Durban, South Africa; 4 Department of Behavioral and Social Sciences, School of Public Health, University of Ghana, Legon, Accra, Ghana

**Keywords:** Type-2 diabetes, Self-care practices, Illness perception, Diabetes knowledge

## Abstract

**Background:**

Self-care practices among persons living with type-2 diabetes are very crucial in diabetes manages as poor self-care results in complications. However, little research exists within the Ghanaian context. This study examined whether type-2 diabetes patients’ illness perception and diabetes knowledge significantly predict diabetes self-care practices.

**Methods:**

A cross-sectional survey design was employed and a total of 160 participants (45 males and 115 females) were sampled from a general hospital in Accra. A self-administered questionnaire measuring illness perception, diabetes knowledge and diabetes self-care practices as well as demographic checklist were used collect data.

**Results:**

Results showed that illness perception and diabetes knowledge significantly predicted overall diabetes self-care practices. Analysis of domain specific self-care practices showed that patients’ diet was significantly predicted by illness perception and diabetes knowledge. Exercise was significantly predicted by only illness perception while blood sugar testing and diabetes foot-care were significantly predicted by diabetes knowledge.

**Conclusion:**

Cognitive and emotional representation of diabetes and diabetes knowledge are key determinants of patients’ diabetes self-care practices. It is therefore important that appropriate psychosocial interventions are developed to help patients’ adherence to recommended self-care practices.

## Background

The prevalence of chronic non-communicable diseases is on the increase within the Sub-Saharan region with its attendant health, economic and social problems [1, 2]. The rate of type-2 diabetes is said to be high in Africa with most cases remaining undiagnosed [1]. The case is not different in Ghana as a developing country where there is a significant diabetes burden [2]. The situation requires identification of possible intervention measures from multiple levels to provide optimum healthcare to persons who are already living with diabetes.

Effective self-management of type-2 diabetes mellitus (T2DM) is crucial to reduce the risk of diabetes-specific complications, such as hypertension, amputation, nephropathy, neuropathy, retinopathy, cardiovascular disease, impotence, and skin lesions [1, 3]. Self-management activities include adherence to diet and nutrition advice, physical activity, taking mediations as prescribed, and weight and stress management [4, 5]. Evidence suggests that there is poor adherence to self-care practices among diabetes patients globally [6]. Thus, it has become necessary to identity factors that play significant roles in influencing the T2DM patients’ adherence to self-care practices.

Illness perception has been identified in some studies as a significant factor that influences self-care practices, psychological distress and other health outcomes among persons living with T2DM [7–9]. The association between illness perception and the health outcomes could be due to the fact that engagement in self-care practices involves complex decision making which depends on the patients’ representation of their illness in terms of whether it is controllable, comprehensible, curable, cyclical and severe or not. However, it has been observed among a sample of Ghanaians living with diabetes that their overall illness perception influences their level of psychological distress [10]. Further evidence has suggested a strong link between diabetes perception and self-care practices [11–13]. For example, Broadbent et al. [12] found that patients’ diabetes perceptions influence their adherence to medication, diet and exercise. Similarly, illness perception domains predict self-management practices of individuals living with diabetes in the UK [14].

Diabetes knowledge has been identified as one of the key determinants of adherence to diabetes self-care practices. This association is very crucial to diabetes intervention as both diabetes knowledge and self-care practices are significantly associated with glycemic control- a measure of diabetes outcome [4, 15–18]. Knowledge about the illness is likely to inform patients about specific actions in the diabetes management process. Thus, the more knowledge patients have about their illness, the more likely they are to comprehend their illness and take up self-care behaviors such as diet, exercise and blood sugar testing among others.

Diabetes health literacy which is an indication of knowledge has been shown to affect self-care practices among persons living with diabetes [19]. In their study, van der Heide et al. [19] examined the mediating role of diabetes knowledge on the relationship between diabetes health literacy and self-care practices found that lower health literacy was significantly associated with less diabetes knowledge, higher glycated hemoglobin (HbA1c) level, less self-control of glucose level, and less physical activity. The same study also found that patients with more diabetes knowledge were less likely to smoke and more likely to control glucose levels, and that diabetes knowledge mediated the association between health literacy and glucose self-control and between health literacy and smoking [19]. This study emphasized the importance of diabetes knowledge in self-care practices as it demonstrated both direct and indirect effects on self-care practices. Previous studies conducted with persons living with diabetes in United States and Netherlands also found possible relationships between diabetes knowledge and self-care behaviors [15, 20].

Despite all these evidence suggesting that the diabetes knowledge has a significant influence on self-care practices and other health outcomes of persons living with T2DM, sparse research exists on how self-care practices of Ghanaians living with T2DM are influenced by their illness perception and diabetes knowledge. Furthermore, findings of the influence diabetes knowledge and illness perception on self-care practices as reported from developed countries cannot necessarily be adduced to diabetes patients living in Ghana. This study was therefore conducted to examine whether self-care practices of persons living with T2DM are significantly predicted by their illness perception and diabetes knowledge. The outcomes from this study may inform interventions aimed at promoting optimum health outcomes of persons living T2DM.

Two key models underpin this study, the health belief model and the self-regulation model [21, 22]. These models were used to examine how illness perception and diabetes knowledge influence the self-care practices of persons living with type-2 diabetes in Ghana. These two models overlap in explaining self-care practices, however, the self-regulation model [22] was used to explain how illness perception influence diabetes self-care among the participants. The self-regulation model developed by Leventhal, Meyer and Nerenz [22, 23] posits that when individuals are faced with threatening situations like chronic illnesses, they tend to generate both emotional and cognitive representations of the illness which influence their actions regarding the threating situation [23]. According to the proponents of this theory, individuals with living with chronic illnesses including diabetes develop several perceptual components (e.g. perceptions of illness identity, cause of illness, duration, consequence, and curability or controllability) which influence how patients interpret their illnesses. In applying this theory to the study, we hypothesized that illness perception could be negatively associated with the various components of self-care practices of persons living with type-2 diabetes.

## Methods

### Design and sample

This study was based on a cross-sectional design which allowed the researchers to collect data from a sample within a particular a population at any one point in time. This design was appropriate as this study was examining relationships and predictive in nature [24]. All persons living with T2DM and receiving health care at a public health facility in Accra, Ghana constituted the population for the study. This is one of the largest primary care hospitals in Accra that provides care for diverse groups of patients on daily basis. A total of one hundred and sixty (160) participants were conveniently sampled for the study. The participants were sampled and included in the study if they met the following inclusion criteria: (1) aged 30 years and above; (2) have been living with diabetes for a year or more; (3) willing to give informed consent or assent to participate in the study.

### Measures

#### Self-care practices questionnaire [25]

This is an 11-item self-report questionnaire which consists of five domains of diabetes self-care practices. These domains include *diet* measured by four items (e.g. How many of the last seven days have you followed a healthful eating plan?), *exercise* measured by two items (e.g. On how many of the last 7 days did you participate in at least 30 min of physical activity), *blood sugar testing* measured by two items (e.g. On how many of the last 7 days did you test your blood sugar?), *foot care* measured by two items (e.g. On how many of the last seven days did you check your feet?) and the final subscale which is *medications* was measured by one item (on how many of the last 7 days, did you take your recommended diabetes medication?). These subscales were measured on a seven-point Likert response format raging between 0 and 7. The mean scores on the items were computed for the first four scales to obtain the subscale scores each domain. The sum of the subscales creates a composite score for total self-care. An overall internal consistency value (α) of .76 was obtained for this study.

#### Diabetes knowledge test [26]

This is a 24-item test which measures diabetes knowledge among respondents. Each question has a correct response and two wrong responses (True, False and Don’t Know). One mark was awarded for each correct response and the total possible score for each respondent ranged between 0 and 24 with higher scores reflecting higher diabetes knowledge. Some of the items on the test include; “Eating too much sugar and other sweet foods is a cause of diabetes”, “The usual cause of diabetes is lack of effective insulin in the body”. A Cronbach alpha of .72 was found for the present the study.

#### Brief illness perception questionnaire [27]

This questionnaire has 9-items which measure patients’ cognitive and emotional representations of their illness with regards to their perceptions of illness consequences, duration, personal control, treatment control, symptoms, coherence, concern, emotional response, and causes. Examples of items on the scale include; how much control do you feel you have over your illness? The causal item was open ended to allow respondents to indicate what they thought caused their illness but was not used in this study. A total illness belief or perception score was computed by adding the responses on all the eight scales with possible scores ranging between 0 and 80. A higher score indicates a more threatening view about diabetes, that is, the individual perceives the illness to be fatal and dangerous to one’s survival. A Cronbach alpha of .78 was found for the present the study.

### Procedure

Ethical clearance was obtained from the Greater Accra Regional Health Directorate of the Ghana Health Service. The clearance letter and the proposal for this study were sent to the head of the La General Hospital to seek permission to use the hospital as the study site. After the head of the facility gave the permission for the data collection, the patients receiving diabetes care at the outpatients department of the diabetes unit diagnosed with T2DM were approached. The objectives of the study were explained to the patients and those who agreed to participate in the study gave consent or assent. The questionnaires were self-administered or interviewer-administered depending on the level of education of the participants. It took an average of 25 min for a questionnaire to be completed. The completed questionnaires were retrieved from the participants on the same day for coding and analysis. The entire data collection was between January and February, 2015. No incentive was provided as participation in the study was voluntary.

### Ethical considerations

All the ethical guidelines concerning the use of human participants in research (e.g. informed consent, confidentiality, privacy, no harm, voluntary participation etc.) were strictly adhered to in the research process. Ethical clearance was obtained from the Greater Accra Regional Health Directorate of the Ghana Health Service which is responsible for healthcare administration within the Greater Accra region of Ghana.

### Data analyses

Data was analyzed using the Statistical Package for the Social Sciences version 21.0 for Window (IBM SPSS). To determine the best predictors of self-care practices, two analyses were conducted. First, the Pearson-moment correlation coefficient (*r*) was conducted to examine the relationship between the self-care practice and its domains (diet, exercise, BST, foot care and medications), illness perception and diabetes knowledge. Secondly, regression fitted models were run using total self-care and five domains as outcome variables. Only predictors that had significant correlation coefficients with the outcome variables entered into the regression models. We thus conducted simple regression models where only one variable was associated with any of the outcome variables and multiple regressions when two or more variables were associated with outcomes variables. All test conducted were two-tailed and held statistical significance at *p* < .05.

## Results

### Demographic characteristics of the sample

The demographic characteristics of the sample are presented in Table 1. Males comprised approximately 72% of the sample, and approximately 92% of the participants were over 40 years old (*Mean* = 60.3 years; *SD* = 12.04). Only 5% of the participants were single, with 39.4% currently married and 28.7% widowed. The majority of the participants had junior secondary education (36.3%) and high secondary education (23.1%), and about over a third of the participants (33.8%) have lived with diabetes for a period of 1–5 years. One average participant has had diabetes for approximately 10 years. The majority of the participants were Christians (87.5%) with the remaining 12.5% representing Islam, African Traditional Religion (ATR) and other religions.

**Table 1:** Background characteristics of respondents

Sample characteristics	*N*	*%*
Gender		
Male	45	28.1
Female	115	71.9
Age of respondents (yrs.) (*Mean* = 60.39*, SD* = 12.04)		
30–40	12	7.5
41–50	22	13.8
51–60	38	23.7
61–70	46	28.8
71 and above	42	26.2
Marital status		
Single	8	5.0
Married	63	39.4
Divorced	16	10.0
Separated	27	16.9
Widowed	46	28.7
Level of education		
No formal education	15	9.3
Primary	28	17.5
Junior secondary	58	36.3
Senior high	37	23.1
Tertiary	22	13.8
Religion		
Christianity	140	87.5
Islam	12	7.5
African Traditional Religion (ATR)	2	1.3
Others	6	3.7
Duration of diabetes (years) (*Mean* = 9.96, *SD* = 7.25)		
1–5	54	33.8
6–10	41	25.6
11–15	33	20.6
16 years and above	32	20.0

*N* Number, *SD* standard deviation

### Relationship between diabetes self-care practices and other study variables

The Pearson correlation analysis was performed to examine the relationship between diabetes self-care practices, illness perception and diabetes knowledge. The results from the analysis as Table 2 showed that there is a significant negative relationship between diabetes self-care practices and illness perception (*r* = −.41, *p* < . 001). It was also observed that a statistically significant positive relationship exists between diabetes self-care practices and diabetes knowledge (*r* = .31, *p* < .001). These results suggest that when patients perceived their illness to be threatening, they engage in less self-care practices but when patients have higher diabetes knowledge, they engage in more diabetes self-care practices.

**Table 2:** Correlation matrix showing the relationship between diabetes self-care practices and other study variables

	Variables	1	2	3	4	5	6	7
1	Self-care	1						
2	Diet	.83***	1					
3	Exercise	.59***	.39***	1				
4	BST	.52***	.33***	−.11	1			
5	Foot-care	.70***	.40***	.07	.57***	1		
6	Medication	.32***	.26**	.14	.10	.05	1	
7	Perception	−.41***	−.33***	−.57***	.02	−.08	−.10	1
8	Knowledge	.31***	.46***	−.10	.43***	.18*	.03	−.01

* *p* < .05

** *p* < .01

*** *p* < .001

The results as presented in Table 2 further showed that patient’ dietary practices were significantly and negatively correlated with illness perception (*r* = −.33, *p* < .001) but positively correlated with diabetes knowledge (*r* = .46, *p* < .001). Results also showed that exercise was significantly and negatively correlated with illness perception, (*r* = −.57, *p* < .001). However, patients’ diabetes knowledge was significantly and positively correlated with both blood sugar testing (BST) (*r* = .43, *p* < .001 and foot-care (*r* = .18, *p* < .05). Adherence to medication was not associated with both illness perception and diabetes knowledge.

### Predictors of diabetes self-care practices

To determine the predictive effect of the illness perception and diabetes knowledge on patients overall self-care practices and domains of self-care practices, single and multiple regression analyses were conducted. The results presented in Table 3 showed that in the first model, illness perception and diabetes knowledge jointly predicted overall diabetes self-care practices, (*R* ^*2*^ = .26, *F* = 27.05, *p* < .001), explaining 26% of variance in the overall diabetes self-care practices.

**Table 3:** Predictors of diabetic self-care practices

Criterion variables	Predictors	Collinearity statistics					
		*B*	*SEB*	β	*t*	*R* ^*2*^	*F*
1. Self-care	Perception	−.74	.13	−.40	−5.85***	.26	27.05***
	Knowledge	1.23	.28	.30	4.40***		
2. Diet	Perception	−.25	.05	−.33	−4.97***	.32	37.26***
	Knowledge	.79	.11	.46	7.02***		
3. Exercise	Perception	−.43	.05	−.57	−8.80***	.34	39.83***
4. BST	Knowledge	.35	.06	.43	6.01***	.19	18.12***
5. Foot-care	Knowledge	.28	.12	.18	2.27*	.04	3.03*

*B* unstandardized coefficient beta, *SE B* standard error of *B*, β standardized coefficients beta

* *p* < .05

** *p* < .01

*** *p* < .001

An examination of the subscales from the Table 3 showed that the model 2 significantly predicted diet (*R* ^*2*^ = .32, *F* = 37.26, *p* < .001). This means that illness perception and diabetes knowledge jointly explained 32% of variance in dietary practices of T2DM patients in the study. Further, the result from the model 3 showed that only illness perception significantly predicted exercise among T2DM patients explaining 34% of variance in exercise behavior (*R* ^*2*^ = .34, *F* = 39.83, *p* < .001). The fourth model revealed that blood sugar testing was significantly predicted by only diabetes knowledge explaining 19% of variance in their blood sugar testing (*R* ^*2*^ = .19, *F* = 18.12, *p* < .001). The fifth model showed that foot-care was significantly predicted by diabetes knowledge explaining only 4% of variance in foot-care practices among T2DM patients (*R* ^*2*^ = .04, *F* = 3.03, *p* < .05).

## Discussion

This study sought to investigate whether illness perception and diabetes knowledge of persons living with type-2 diabetes have any significant on their overall self-care practices and domain specific practices. Self-care practices among persons living with type-2 diabetes have been associated with better diabetes outcomes and as such, identification of variables that significantly predict these self-care practices is very paramount within our Ghanaian context. Results from the analysis showed that illness perception significantly predicted overall self-care practices among persons living with type-2 diabetes. That is, when patients hold more threatening views about their illness, they tend to engage in less diabetes self-care practices. This could be due to that when patients view their illness to be more threatening, they may adopt a fatalistic view and thus, do not place much value on their own personal recovery in the management of their illness.

This finding is consistent with previous findings that showed that diabetes perception significantly influenced self-care practices among persons living with type-2 diabetes [7, 9]. The predictive effect of illness perception on diabetes outcomes have also been documented within the Ghanaian context with evidence suggesting that illness perception significantly predict the levels of general psychological distress, depression and anxiety levels of persons living with diabetes [8, 10]. The impact of illness perception is not limited to the overall illness perceptions as other researchers have found specific domains such as timeline, controllability, severity and illness coherence to predictive of diabetes self-care practices among a sample of diabetic patients in developed countries [14].

Further analysis of the predictive effects of illness perception on the domain specific diabetes self-care practices showed that illness perception only predicted diet and exercise practices among persons living with type-2 diabetes which is partially consistent with earlier findings by Broadbent et al. [12] that patients’ diabetes perceptions influence their adherence to medication, diet and exercise significantly. The findings from the current findings have implications for the involvement of psychologists in the delivery of diabetes care as the cognitive and emotional representation of the illness by the patients could pose a great challenge to their engagement in diabetes self-care practices especially within the dietary and exercise domains.

The effect of diabetes knowledge on the overall self-care practices and domain specific diabetes self-care practices were also examined and the results from the analysis showed that diabetes knowledge significantly and positively predicted overall diabetes self-care practices. This shows that the more knowledge diabetic patients have about their illness, the more likely they are to engage in diabetes self-care practices. This finding is consistent with previous results that found diabetes knowledge to be predictive of diabetes self-care practices and diabetes management [15, 16, 19]. The findings regarding the predictive effects of diabetes knowledge on the domain specific diabetes self-care practices showed that diabetes knowledge significantly and positively predicted diet, blood sugar testing and foot-care practices. These findings could be attributed to the fact that engagement in these domain specific diabetes self-care practices requires deep knowledge about diabetes and the relevance of these specific practices to the management process unlike medication that is widely known to influence illness outcome. The outcomes are consistent with the previous works by van der Heide et al. [19] which found diabetes knowledge to be significantly associated with domain specific diabetes self-care practices.

The findings from this study have some practical implications for diabetes healthcare delivery. Firstly, the significant associations found between the predictor variables (illness perception and diabetes knowledge) and diabetes self-care practices suggests that there is the need to train healthcare providers (nurses) to address the chronic care needs of persons with diabetes. Secondly, nurses and doctors should be equipped with resources to address the self-care needs of patients with diabetes in the form of information leaflets and other relevant materials to aid independent self-care. Such self-care information should include information on causes, prognosis and management strategies. Finally, since group education is generally practiced at the various diabetes units, it is recommended that individual needs should also be taken into consideration due to variations in educational levels and interpretation of the educational materials.

The findings of this study should be interpreted cautiously in the light of some limitations. The use of a cross-sectional survey design does not allow for the generalization of the findings beyond the sample from which data was gathered. The study did not also consider impacts of personal demographic characteristics on adherence to diabetes self-care practices neither was the perceived causes of the illness measured. It is also worth noting that the relationships examined do not reflect diabetes control since no blood samples were analyzed for HbgA1c or other physiological measures. Finally, the collected data was based on self-report, and may therefore, not be consistent with complications from a medical point of view. However, it has been argued that that perceived health and illness are often considered to be more predictive of health behaviours and outcomes than objective, medical indicators [28]. Despite these limitations, this study has provided the foundation for future empirical studies among persons living with diabetes in relation to their diabetes management. Future study may use qualitative design to understand the key motivations to engage in diabetes self-care practices in terms of barriers and facilitators.

## Conclusion

The findings from this current study suggest that diabetes self-care practices of persons living with type-2 diabetes in Ghana are significantly influenced by their illness perception and their level of diabetes knowledge. Therefore, measures should be put in place to increase patients’ level of knowledge and also, healthcare practitioners should understand that how patients interpret their illness is very significant in determining whether they will follow the recommended self-care practices or not. The findings from this study add to the existing scanty literature on diabetes self-care practices within the Ghanaian context as this study provides evidence that could be culturally relevant to persons living with type-2 diabetes in Ghana.

## Abbreviations

BST: blood sugar testing
SD: standard deviation
SPSS: Statistical Package for the Social Sciences
T2DM: type-2 diabetes mellitus
